# Mapping and Preliminary Analysis of ABORTED MICROSPORES (*AMS*) as the Candidate Gene Underlying the Male Sterility (*MS-5*) Mutant in Melon (*Cucumis melo* L.)

**DOI:** 10.3389/fpls.2017.00902

**Published:** 2017-05-30

**Authors:** Yunyan Sheng, Yudan Wang, Shiqi Jiao, Yazhong Jin, Peng Ji, Feishi Luan

**Affiliations:** ^1^Department of Agriculture, Heilongjiang Bayi Agricultural UniversityDaqing, China; ^2^Key Laboratory of Biology and Genetic Improvement of Horticultural Crops (Northeast Region), Ministry of Agriculture/Northeast Agricultural UniversityHarbin, China; ^3^Department of Horticulture, Northeast Agricultural UniversityHarbin, China

**Keywords:** melon, genetic male sterility, fine mapping, SLAF-seq, AMS gene

## Abstract

Melon is an important agricultural and economic vegetable crop worldwide. The genetic male sterility mutant (ms-5) has a recessive nuclear gene that controls the male sterility germplasm. Male sterility could reduce the cost of F_1_ seed production in melon, but heterozygous fertile plants should be removed before pollination. In this study, bulked segregant analysis combined with specific length amplified fragment sequencing was applied to map the single nuclear male sterility recessive gene. A 30-kb candidate region on chromosome 9 located on scaffold 000048 and spanning 2,522,791 to 2,555,104 bp was identified and further confirmed by cleavage amplified polymorphic sequence markers based on parental line resequencing data and classical mapping of 252 F_2_ individuals. Gene prediction indicated that six annotated genes are present in the 30-kb candidate region. Quantitative RT-PCR revealed significant differences in the expression level of the LOC103498166 ABORTED MICROSPORES (*AMS*) gene in male-sterile lines (ms-5) and male-fertile (HM1-1) lines during the 2-mm (tetrad) and 5-mm (the first pollen mitosis) periods, and negative regulation of the *AMS* candidate gene transcription factor was also detected. Sequencing and cluster analysis of the *AMS* transcription factor revealed five single-nucleotide polymorphisms between the parental lines. The data presented herein suggest that the *AMS* transcription factor is a possible candidate gene for single nuclear male sterility in melon. The results of this study will help breeders to identify male-sterile and -fertile plants at seeding as marker-assisted selection methods, which would reduce the cost of seed production and improve the use of male-sterile lines in melon.

## Introduction

Compared with other horticultural plants, melon (*Cucumis melo* L.) exhibits considerable hybrid vigor (heterosis). Male sterility may help breeders reduce the labor demand and cost of F_1_ production (Park et al., 2004). Male sterility in higher plants is caused by mitochondrial genes along with certain nuclear genes, resulting in cytoplasmic male sterility (CMS). The male sterility, occurrence and restoration of fertility of CMS is under control of the interaction between mitochondrial and nuclear genes. It shows non-Mendelian inheritance, with male sterility inherited maternally. In general there are two types of cytoplasm: N (normal) and aberrant S (sterile) cytoplasms. CMS-based hybrid seed technology uses a three line system, the CMS lines, the maintainer lines, and the restore line (Chen and Liu, 2014; Zhang et al., 2017). Male sterility from a single nuclear gene results in the condition known as genetic male sterility (GMS) (Li et al., 1985; Wang et al., 2013; Chen and Liu, 2014). In addition, genes that participate in stamen development, archesporial cell differentiation, meiosis, microspore nucleus mitosis and pollen differentiation can induce the development of unusual buds that result in male sterility (Ma et al., 2012). GMS is a stable and effective means to reduce the high cost and increase the quality and quantity of seed in hybrid F1 production. GMS also provides important materials for studying pollen and flower development. Therefore, male sterility is a cutting-edge and important research area in higher plants. GMS is a stable and complete form of male sterility, and the male sterility genes of GMS plants are easily transferrable (Su et al., 2016). The major drawback of GMS is that the offspring of GMS plants pollinated by heterozygous male-fertile-plants always segregate in a 1:1 ratio; this segregation requires the eradication of male-fertile plants from the maternal line (Huang et al., 2007). The GMS genetic control mechanism can be exerted by a single recessive gene (Huang et al., 2007), by double recessive genes (Tu et al., 1997), or by interacting recessive genes (Chen et al., 1998). Several nuclear recessive genetic male sterility genes have been finely mapped (Wang et al., 2006; Huang et al., 2007; Bartoszewski et al., 2012; Frouin et al., 2014; Pranathi et al., 2016; Zhang et al., 2016). In rice, more than 30 nuclear genes have been mapped (Qi et al., 2014; Sheng et al., 2015), and more than 20 genes have been finely mapped in pepper (Bartoszewski et al., 2012). Several male-sterile tomato mutants have been utilized, including positional sterile 2 (*ps 2*) (Gorguet et al., 2009) and male sterile 10 (*ms 10*) (Panthee and Gardner, 2013). However, few reports have been published on male sterility in melon.

Only five single recessive genes for male sterility—ms-1 to ms-5—have been investigated (Bohn and Whitaker, 1949; Bohn and Principe, 1964; McCreight and Elmstrom, 1984; Lecouviour et al., 1990; Pitrat, 2002; Park et al., 2004). Each of these genes is located on a different chromosome and presents a unique phenotype. The phenotypes of ms-1 and ms-2 are difficult to observe in the field, but ms-3, ms-4, and ms-5 are easily detected by their phenotype. The ms-2 mutant was found during the cultivation of La Jolla 40460 cantaloupe. The mutant material has excellent properties, such as resistance to powdery mildew. The stamens of the mutant plants are smaller than those of the normal-flower plants, and the pollen sac is not cracked in the mutants. Microscopic observations showed that the ms-2 mutant has a small amount of or no pollen; the successful rate of artificial pollination is approximately 12 times lower than that of other plants (Bohn and Principe, 1964). The study of genetic inheritance indicated that the separation rate of the male-fertile and male-sterile plants is 3:1 in the F_2_ population, which carries the mutant hybridization of ms-2, and that the separation rate of the male-fertile and male-sterile plants is 9:7 (Bohn and Principe, 1964). The ms-3 mutant can be identified by its phenotype with the naked eye (McCreight and Elmstrom, 1984). Park et al. (2004) used ms-3 mutants and “TAM Dulce” to study the genetic inheritance of the ms-3 gene, a goodness-of-fit to 3:1 ratio for the number of male fertility to sterile F_2_ plants derived from ms-3 was observed. And found that ms-3 is controlled by a pair of recessive genes; a RAPD marker OAM08.650 with a linkage distance of 2.1 cM was also found. A SCAR marker SOAM08.644 and RAPD marker OAM08.650 were confirmed in another F_2_ population from ms-3 × “Misssion” to be consistently links to *ms-3* gene at 5.2 cM. These results were consistent with those of McCreight (1983), McCreight and Elmstrom (1984) and also showed that *ms-L* and male sterile-Leesburg are the *ms-3* gene. The male flower of ms-4 and ms-5 plants degraded at the early stage of floral organ development, making it easy to identify these phenotypic characteristics in the field (Lecouviour et al., 1990; Pitrat, 1991). The ms-5 mutant was first discovered by the Clause Seed Company in the United States (Lecouviour et al., 1990). This mutant was first discovered in 1966 during cultivation of powdery mildew-resistant material “PMR45.” The male flowers of mutant plants during the early stage of bud development are significantly less developed than those of fertile plants; male flowers of complete plants have a few or no anthers. Previous studies have shown that *ms-3*, *ms-4*, and *ms-5* are all male sterility genes that are inherited independently (McCreight and Elmstrom, 1984; Lecouviour et al., 1990). These genes are located on different chromosomes in the melon traditional genetic map (Pitrat, 1991, 2002). The andromonoecious gene (*a*) in melon controls the development of the stamens in female flowers (Boualem et al., 2008). Park’s results confirmed that the (*a*) gene and *ms-3* gene were not genetically linked, and it was also shown that the (*a*) gene is not genetically linked to the other male sterility genes (Pitrat, 1991, 2002). More recently, the inheritance of ms-5 was investigated by our lab, and the results indicated that the segregation ratio of fertile and sterile plants in the F_2_ generation was 3:1, the segregation ratio of P_1_BC_1_ (P_1_ × F_1_, P_1_ is male fertile) male fertility and sterility was 1:1, and all P_2_BC_1_ (P_2_ × F_1_, P_2_ is male fertile) offspring were male fertile. These results were consistent with previous results that demonstrated that ms-5 is controlled by a single recessive gene (Lecouviour et al., 1990; Pitrat, 1991; Park et al., 2004).

Next-generation sequencing (NGS) technology makes it possible to obtain thousands of single-nucleotide polymorphisms (SNPs) throughout the entire genome for rapid identification of target genes or QTL regions (Zhao et al., 2016; Zheng et al., 2016). Since the first high-resolution genetic map was developed by large-scale *de novo* SNP(Single Nucleotide Polymorphism) marker discovery using the specific length amplified fragment (SLAF) approach in *Cyprinus carpio* L. (Sun et al., 2013), advanced NGS technology has offered new strategies for identifying quantitative and qualitative traits (Qi et al., 2014; Xu et al., 2015). Bulked segregant analysis (BSA) is an efficient and rapid method for identifying molecular markers linked to special traits using bulk DNA from F_2_ plants (Takagi et al., 2013). After the first application of NGS and BSA for the identification of plant height QTLs in rice (Ikeda et al., 2013), through a combination of NGS and BSA methods, the tagging of quality and quantity genes has been conducted in numerous species; such tagging has involved branch numbers in rice (Chen et al., 2015) and maize (Xia et al., 2015), *Flavor Contributing Traits* in melon (Zhang et al., 2016), and seed weight in *Brassica napus* (Geng et al., 2016) as well as the blast resistance gene in rice (Zhang et al., 2016) and *Tssd* locus in peach (Lu et al., 2016). More recently, BSA combined with SLAF-seq was used to finely map the gene controlling fruit flesh thickness in cucumber (Xu et al., 2015). These methods, using distinct or opposing extreme phenotypes, can directly map target genes without laborious, time-consuming work.

In this study, we applied this approach to F_2_ populations in combination with genome sequencing of parental lines to rapidly map and characterize the ms-5 gene in melon. The ABORTED MICROSPORES (*AMS*) gene was identified as a candidate gene for male sterility. The results will help breeders identify male-sterile plants in seedling and will increase melon breeding program, especially with regard to using male sterility to breed new germplasm and increasing yields of high-quality melon.

## Materials and Methods

### Plant Materials and Cultivation

Two hundred fifty-two F_2_ plants and F_2:3_ families were derived from the melon cross of male-sterile line ms-5×HM1-1 for identification of the male sterility gene in the greenhouse of Heilongjiang Bayi Agricultural University in 2014 and 2015. The male-sterile mutant ms-5 was discovered in attempts to breed powdery mildew resistance from ‘PMR45’ into the variety Charentais (McCreight and Elmstrom, 1984). The ms-5 mutant was easily selected in the greenhouse by its phenotype due to male flower abortion at the bud stage (Lecouviour et al., 1990; Pitrat, 1991). The male-fertile ‘HM1-1,’ which produces a thin-skinned melon, from the northeast area of China, is an andromonoecious line with early flowering time. The F_2_ population for primary mapping consisted of 252 plants and was obtained by selfing F_1_ plants grown in the greenhouse of the experimental station at Heilongjiang Bayi Agricultural University (45°N, 125°E), Heilongjiang Province, China, in the summer of 2014. To distinguish homozygous and heterozygous fertile F_2_ plants, 189 F_3_ families were derived from selected fertile F_2_ plants (15 plants per family for F_2_ plants; of all 252 F_2_ plants, 63 were male sterile and 189 male fertile). Homozygous F_2_ plants were identified by investigation of F_3_ families. Each F_3_ family included 15 plants. When all plants from each family showed male fertility in the field, it was considered a male-fertile homozygous family; if an F_3_ family contained male-sterile and male-fertile plants, it was considered a heterozygous family. One hundred seventy-one P_1_BC_1_ and 120 P_2_BC_1_ plants were planted in the open field in 2015.

The chi-square test was used to identify significant differences in the segregation ratio in F_2_, P_1_BC_1_ and P_2_BC_1_.

### Fertility Characterization

Due to the ease of investigating flower pollen fertilization, visual observation and a light microscope were used to assess fertility. Pre-flowering buds were collected and dipped into 1% (v/v) acetocarmine. Plants were considered completely male sterile if the pollen did not stain, and plants exhibiting more than 90% darkly stained pollen were considered fertile.

### DNA Preparation

Two bulks for sequencing were prepared by selecting individuals from the F_2_ population of 252 plants; 30 individual plants that were consistently homozygous fertile and 30 sterile plants from the F_2_ generation were selected. Genomic DNA was extracted from tender leaves of each individual using a modified CTAB method. Each bulk was made by mixing equal amounts of DNA from the 30 homozygous fertile plants or sterile plants. DNA quality and concentration were measured by 1% agarose gel electrophoresis, and the final DNA concentration was adjusted to 100 ng/μL.

### Whole-Genome Sequencing of Parental Lines and Bulks via SLAF–seq

Two bulked pools and parental lines (ms-5 and HM1-1) were sequenced on an Illumina GaIIx machine, Biomarker, Beijing, China. The procedures for SLAF library preparation and sequencing were performed as described by Sun et al. (2013) with minor modifications. According to Biomark’s sequencing instructions, genomic DNA from parental lines and from the two bulked pools was incubated at 37°C with 0.6 U MseI (New England Biolabs, Hitchin, Herts, United Kingdom), T4 DNA ligase (NEB), ATP (NEB) and MseI adapters. Restriction-ligation reactions were heat inactivated at 65°C, after which the samples were digested in an additional restriction process with the enzymes HaeIII and BfaI at 37°C. The diluted restriction-ligation samples, dNTPs, Taq DNA polymerase (NEB), and MseI primer were used for PCR. The PCR products were purified using an ENZAH cycle pure Kit (Omega) and pooled.

The samples were incubated at 37°C with MseI, T4 DNA ligase, ATP and Solexa adapters; purified using a Quick Spin column (Qiagen); and run on a 2% agarose gel to isolate 260–420-bp fragments using a gel extraction kit (Qiagen). These fragments were used in PCR amplification with Phusion Master Mix (NEB) and Solexa amplification primer mix. Phusion PCR settings followed the Illumina sample preparation guide. Samples were gel purified, and products with appropriate sizes (260–420 bp) were excised and diluted for sequencing using the Illumina GAIIx (Illumina, San Diego, CA, United States).

SLAF_Poly.pl (Biomarker, Beijing, China) was used to analyze the massive sequences obtained. Sequences from a BLAST comparison with greater than 90% identity were grouped into one SLAF locus, and 260–420-bp fragments were selected for molecular marker development. The selected reads were compared to the reference genome^1^. Polymorphic SLAFs between two parents and markers that showed polymorphism between bulked pools were summarized and compared among the parental lines and pools.

### Association Analysis

Pair-end sequencing was performed according to the selected SLAFs using an Illumina high-throughput sequencing platform, followed by SNP genotyping and evaluation. Analysis of SLAF-seq data was conducted according to the methods of Abe et al. (2012). SLAF single-end reads were grouped based on sequence similarity, as detected by BLAT (Kent, 2002). SLAFs with two, three, or four tags were considered to be polymorphic markers. The SNP index indicates the proportion of reads harboring an SNP that is different from the reference sequence. The parameters SNP-index and Δ(SNP-index) (Abe et al., 2012; Takagi et al., 2013) were calculated to identify candidate regions for melon male sterility.

In this study, P indicates the female parent (sterile), M indicates the male parent (fertile), aa indicates the sterile pool, and ab indicates the fertile pool. Δ(SNP_index) = SNP_index(aa) – SNP_index(ab). SNP_index(ab) = Mab/(Pab + Mab), where Mab indicates the depth of the ab population derived from M and Pab indicates the depth of the ab population derived from P. Furthermore, SNP_index(aa) = Maa/(Paa + Maa), where Maa indicates the depth of the aa population derived from M and Paa indicates the depth of the aa population derived from P. The Δ(SNP-index) = 1 if the bulked DNA comprises only the parental HM genome, the Δ(SNP-index) = -1 if it is of the parent ms-5 genome only, and the Δ(SNP-index) = 0 if both parents have the same SNP indices at the genomic regions. An SNP with a Δ(SNP-index) > 0.6 indicates linkage with the male sterility locus.

### Development of SNP Markers Based on SLAF-seq

To minimize the genetic interval for fine mapping and to validate the SNP markers based on SLAF-seq, cleavage amplified polymorphic sequence (CAPS) markers were developed (Supplementary Table S1). Primers for CAPS markers were designed with Primer Premier 5.0 (Premier Biosoft International, Palo Alto, CA, United States^2^) and CAPS Finder 2.0^3^. Each PCR contained 30 ng of template DNA, 1.0 μM each of forward and reverse primers, 0.2 mM of dNTP mix, 0.1 U of Taq DNA polymerase, and 1 × PCR buffer (Takara, China) in a total volume of 10 μL. Restriction enzymes were added to the PCR reaction after performing the specific primer-based PCR program and incubation for 2 h at the temperature according to the manufacturer’s instructions. A 6% polyacrylamide gel with silver staining was used to separate the digested products.

### Linkage Map Construction and ms-5 Gene Mapping

Markers were screened first among the ms-5, HM1-1 and F_1_ plants. Linkage analysis was then performed for the polymorphic markers in the F_2_ generation, which consisted of 252 plants. F_2_ population data were collected for genetic mapping analysis. F_2_ fertile plants phenotype screening was based on the F_3_ family (each F_3_ family consisted of 15 plants). For genotype a, the F_2_ progeny were all male sterile, they were not testing for F_3_ families. For genotype b, the F_3_ family consisted of male-fertile plants. For genotype H, the F_3_ family consisted of male-fertile and male sterile plants. JoinMap 4.0 was used to develop linkage groups by association region. The Kosambi map function was used to calculate the genetic map distance between markers.

### qRT-PCR

Total RNA was isolated from flower buds of male-sterile (ms) plants from ms-5 and male-fertile (fs) plants from HM1-1, 20 homozygous male-sterile F_2_ plants and male-fertile F_2_ plants were pooled, and RNA was extracted for candidate gene expression analysis. Buds of two stages of flower development (2-mm and 5-mm bud diameters; buds ∼2 mm indicate the stage of uninucleated microspore, and buds ∼5 mm indicate the stage of pollen development and maturation, based on Wang et al., 2013; Sheng et al., 2016) were selected for RNA isolation using RNAiso plus (Takara, China). The qRT-PCR reaction was performed using the IQ5 system (Bio-Rad, United States) with 20 μL. The PCR primers were designed using Primer 5.0 software. Details of the primers are shown in (Supplementary Table S2). Each reaction contained 10 μL of 2× TranStart Top Green qPCR Supermix (Transgene, China), 10 pmol of each primer, and 2 μL of cDNA templates, with distilled water added to reach a total volume of 20 μL. The two-set method of the thermal conditions was employed. qRT-PCR was performed using the conditions of 95°C for 15 s and then 55°C for 15 s, followed by a slow increase of temperature by 0.5°C per cycle to 95°C, with continuous measurement of fluorescence. Three replicates were used for the qRT-PCR. The mRNA expression data were collected using the Δ^Ct^ method.

### Sequencing Assembly and DNA Analysis of Candidate Loci

The DNA sequences were retrieved from the NCBI database^4^ and the Cucurbitaceae database of the Weng Lab at the University of Wisconsin-Madison, Madison, WI, United States^5^, using Softberry to predict candidate gene structures and using the Open Reading Frame Finder FGENESH^6^. Multiple nuclear sequence alignment was used DNAMAN6.0 software.

## Results

### Inheritance of the Male Sterility 5 (ms-5) Gene in Melon

**FIGURE 1:**
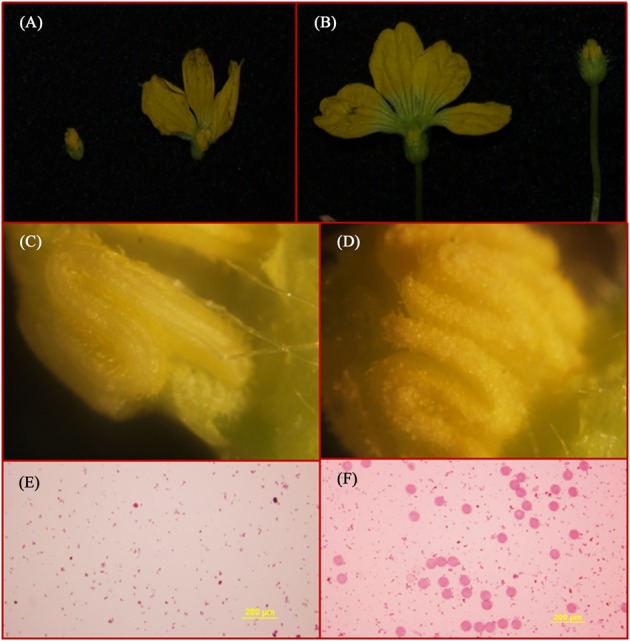
Flower structure and SEM (scanning electron microscopy) of ms5 and HM1-1. **(A)** ms5 is shown on the left with a black background. **(B)** HM1-1 is shown on the right. **(C)** Pollen of ms5 is bright yellow with smooth flower buds. **(D)** HM1-1 is orange (on the right) with full pollen on the surface of buds. **(E)** Pollen viability of ms5 detected using acetocarmine. **(F)** Pollen viability of HM1-1 detected using acetocarmine.

The segregation distribution of male sterility among test materials in 2 years, spring 2014 and 2015, was observed. In the two parental lines, ms-5 exhibited strong male sterility during both years. HM1-1 showed male fertility, and the HM1-1 pollen ability was greater than 98% according to the carmine acetate dyeing method (**Figure 1**). In spring 2014, 30 F_1_ and 160 F_2_ plants were investigated, and 120 P_1_BC_1_ and 60 P_2_BC_1_ plants were also investigated for male sterility. Among these, all F_1_ plants were fertile; 112 plants were male fertile and 48 male sterile in the F_2_ generation in 2014 (*X*^2^ = 1). Sixty-two of P_1_BC_1_ plants showed male fertility 58 male sterility in 2014 (*X*^2^ = 0.07). In 2015, 86 plants exhibited male fertility, and 85 plants of P_1_BC_1_ exhibited male sterility (*X*^2^ = 0). All of the P_2_BC_1_plants investigated in 2014 exhibited male fertility.

In spring 2015, a large number F_2_ and P_1_BC_1_ generation was investigated for male sterility. One hundred forty-eight F_2_ plants showed male sterility, and 502 among 650 F_2_ plants exhibited male fertility (*X*^2^ = 0.89). For the P_1_BC_1_ generation, the ratio of male sterility to fertility was 85:86. The segregation ratio in the F_2_ generation was 3:1, and that of P_1_BC_1_ was 1:1. As indicated from the chi-square test showing that male sterility 5 (ms-5) was controlled by a single nuclear recessive gene, male fertility was dominant over male sterility (**Table 1**).

**Table 1:** Genetic inheritance analysis of genetic male sterility 5 (ms-5) in different generations.

Generation	Total	Male sterility	Male fertility	Expected segregation	*X*^2^-value
ms5	30	30	0		
HM1-1	30	0	30		
F_1_	30	0	30		
F_2_(2014)	160	48	112	1:3	2.13
F_2_(2015)	650	148	502	1:3	1.72
P_1_BC_1_(2014)	120	58	62	1:1	0.12
P_1_BC_1_(2015)	171	85	86	1:1	0.004
P_2_BC_1_(2014)	60	0	60		

*X_0.01_^2^ = 6.63, X^2^ < X_0.01_^2^ the observational ratio fits the exceptional ratio.*

### SLAF-seq Analysis

**FIGURE 2:**
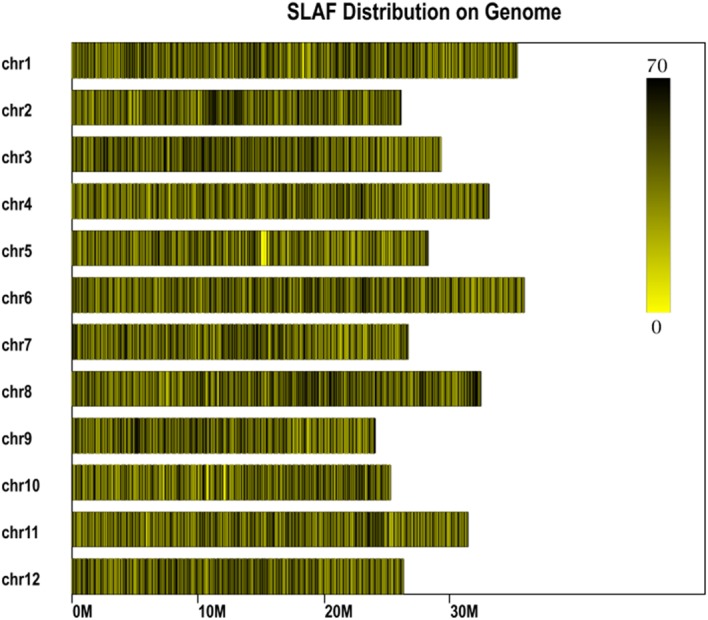
Specific length amplified fragment (SLAF) marker distribution on chromosomes. Abscissa: length of chromosome, each yellow bar denotes one chromosome, and darker color indicates more SLAF markers in each window. ordinate: chromosome ID.

In total, 14.57 million reads were generated after high-throughput sequencing of the constructed SLAF library, with each read being approximately 69 bp in length. Most of these bases (85.08%) were high quality, with quality scores higher than Q20. The GC contents of the sequenced parental lines was 33.18%, on average. The numbers of total reads were 80,896,074 and 87,674,740 for ms-5 and HM1-1, respectively, and the numbers of clean reads of the male and female parents were 40,448,037 and 43,837,370, respectively (**Table 2**). In total, there were 6,779,312 reads of the male-sterile bulk and 7,787,845 of the male-fertile bulk. The average Q30 was 89.18%, and the GC contents of male-sterile and fertile bulks were 37.56 and 38.32%, with an average of 37.94%. The average depth of the SLAF markers was 28.65-fold for ms-5, 30.12-fold for HM1-1, 31.44-fold for the male-sterile bulk and 36.15-fold for the fertile bulk (**Table 2**). After the tags were aligned with the reference genome (**Figure 2**), 108,059 SLAFs were selected for BSA analysis, among which 96,097 tags were polymorphic, with the polymorphic rate of 8.23%. The marker numbers and their assignments on the chromosome are shown in **Table 3**.

**Table 2:** Summary information of the sequencing data.

#Sample	SNP number	Total reads	Q30 percentage (%)	GC content percentage (%)	SLAF number	Total depth	Average depth
ms-5	1,661,769	80,896,074	85.07	35.89	112,432	2,306,812	28.65X
hm1-1	1,004,559	87,674,740	85.08	36.68	112,654	2,523,143	30.12X
Sterility pool	319,824	6,779,312	89.35	37.56	164,751	5,179,771	31.44X
Fertility pool	313,495	7,787,845	89.00	38.32	160,923	5,817,336	36.15X

*SNP number, Single Nucleotide Polymorphism number between samples and melon reference genome.*

**Table 3:** Numbers of SLAF markers on each chromosome in the muskmelon reference genome assembly.

#chr	SLAF	SNP	SNP_on_SLAF	Poly -SLAF	Poly_percent	Length of chromosome
Chr1	15780	2017	824	448	2.84%	39,128,206
Chr2	12059	3265	1365	676	5.61%	28,966,238
Chr3	13837	4264	1685	852	6.16%	32,497,971
Chr4	14205	2596	1051	534	3.76%	36,629,142
Chr5	11753	1754	765	422	3.59%	31,337,173
Chr6	16137	4166	1624	846	5.24%	39,743,896
Chr7	11840	2063	850	435	3.67%	29,607,723
Chr8	15178	3886	1647	870	5.73%	35,954,773
Chr9	10862	5834	4373	2208	20.33%	26,659,220
Chr10	11035	1238	538	286	2.59%	28,046,777
Chr11	13522	2303	1013	522	3.86%	34,770,107
Chr12	12027	3055	1174	618	5.14%	29,194,729
Total	167311	38470	18092	9345	5.59%	450,000,000

*Chr, chromosome number; SALF, SLAF tags number on each chromosome; SNP, SNP numbers in each chromosome; SNP-on -SLAF, SNP number on SALF tags; Poly -SLAF, polymorphism SLAF tags; poly_percent, percentage of polymorphism SLAF tags in all SLAF tags; Length of chromosome, totally length of each chromosome by SLAF sequencing.*

### Association Analysis of Male Sterility

Based on the sequencing criteria of sequence depth in the parents and the origin of male sterility genotype derived from ms-5 and male fertility from HM1-1, 3,870 markers from a total of 9,345 polymorphic SLAF markers were selected for association analysis. According to the results of the ΔSNP index calculation, all selected markers were distributed on chromosome 9. Four regions in melon’s genome—2,628,112 to 3,491,020 (0.86 Mb), 3,838,547 to 4,102,757 (0.26 Mb), 4,304,282 to 6,053,037 (1.75 Mb) and 7,178,780 to 8,599,536 (1.42 Mb)—were regarded as association regions (**Table 4**); in total, 271 genes and 87, 19, 702 and 510 markers, respectively, were located in the candidate regions. To narrow the mapping region, SNPs in the association regions from parental resequencing were analyzed in combination with the bulk F_2_ SLAF-seq data. Twenty-three CAPS (Cleaved Amplified Polymorphic Sequence) markers were designed and used for the 252 F_2_ plants to narrow the association regions, and candidate gene regions for ms-5 on chromosome 9 spanned 4,304,282 to 5,036,784 (**Table 4**). Further analysis was carried out based on the results of the association analysis and gene annotation.

**Table 4:** Information of genetic male sterility association regions.

Chromosome	Start of genome position	End of genome position	Size (Mb)	Gene number
Chr9	2,628,112	3,491,020	0.86	63
Chr9	3,838,547	4,102,757	0.26	14
Chr9	4,304,282	6,053,037	1.75	127
Chr9	7,178,780	8,599,536	1.42	67
Total			4.29	271

### Linkage Map Construction and Candidate Gene Analysis

**FIGURE 3:**
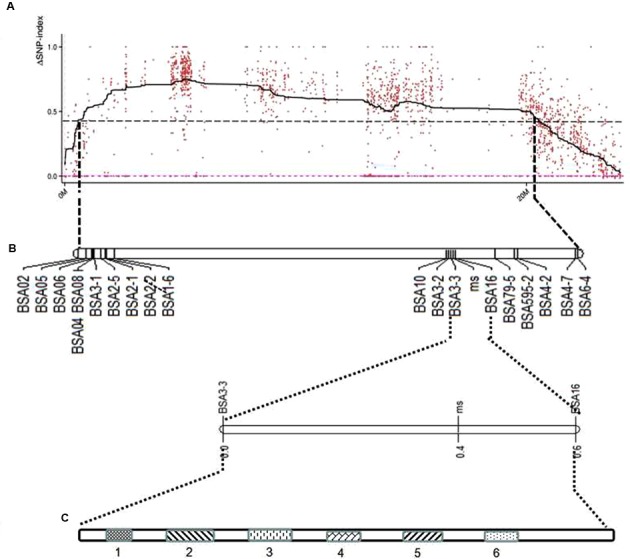
Location of predicted candidate genes on the genetic map of melon chromosome 9 using F_2_ populations. **(A)** ΔSNP polt. The *X*-axis represents the chromosome position, and the *Y*-axis represents the ΔSNP-index value. The black line shows the average values of ΔSNP-index; the red dotted line is the ΔSNP value (0); the threshold value was 4.30, with an imaginary black line, calculated by Loess regression. The peak region contains 22 SLAF markers. **(B)** The genetic map generated using Joinmap 4.0 software. **(C)** Linked markers to male sterility 5 (ms5), and the candidate region is 1.75 Mb; two markers linked to ms5 are BSA3-3 and BSA16, at genetic distances of 0.1 and 0.3 cM, respectively. Fourteen predicted genes were screened, but only six were annotated; the candidate genes are *LOC103498166, LOC103498167, LOC103498168, LOC103498194, LOC103498169*, and *LOC103498170*.

Due to the candidate gene identified on chromosome 9, a linkage map of the association region on chromosome 9 using 252 F_2_ plants with 23 CAPS markers was conducted and the ms-5 gene was mapped. This associated region covered 37.6 cM, with an average distance of 1.88 cM (**Table 2** and **Figure 3**). Finally, 14 genes with ms-5 characters were identified using the Gene Finding program of Softberry^7^. Two markers were linked to ms-5 (BSA16 and BSA3-3, with a genetic distance 0.2 and 0.1 cM, respectively). The marker locations are shown in Supplementary Table S1. Linkage mapping results indicate that ms-5 is located on scaffold 000048 on chromosome 9 from 2,522,791 to 2,555,104, which covers a 32-kb region of the muskmelon line DHL92 reference genome assembly^8^ (Supplementary Table S1). The candidate region spanned 32 kb. Fourteen genes were screened, but only six genes were annotated. In this candidate region, based on gene finding program in softberry^9^ 14 predicted genes sequence were included. After we Blast these 14 genes sequence in NCBI BLast database, only 6 genes were annotated, the other 8 sequences were only the sequence on melon genome. These annotated genes included the *C. melo* transcription factor ABORTED MICROSPORES (LOC103498166), *C. melo* geranylgeranyl transferase type-1 subunit beta (LOC103498167), *C. melo* uncharacterized LOC103498168 (LOC1034981), *C. melo* RING-H2 finger, *C. melo* serine/threonine-protein phosphatase 7-like (LOC103498169), *C. melo* heat shock cognate 70 kDa protein 2-like (LOC103498170), and *C. melo* RING-H2 finger protein ATL52-like (LOC103498194).

### qRT-PCR Analysis

**FIGURE 4:**
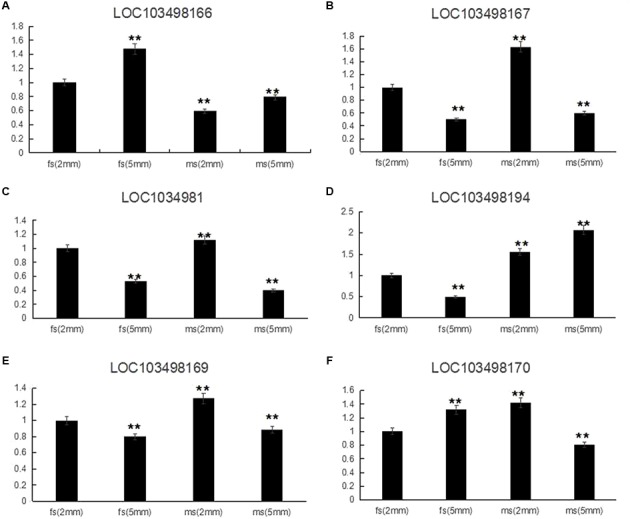
Relative expression of six candidate genes in 2- and 5-mm stage flower buds diameter of ms5 and HM1-1. The melon β-actin gene was used as a control. ^∗∗^Significantly different expression compared with 2-mm buds in fertile plants (HM1-1). **(A)** *Cucumis melo* transcription factor ABORTED MICROSPORES (LOC103498166) gene expression. **(B)** *Cucumis melo* geranylgeranyl transferase type-1 subunit beta (LOC103498167) gene expression. **(C)** *Cucumis melo* uncharacterized LOC103498168 (LOC1034981) gene expression. **(D)** *Cucumis melo* RING-H2 finger protein ATL52-like (LOC103498194) gene expression. **(E)** *Cucumis melo* serine/threonine-protein phosphatase 7-like (LOC103498169) gene expression. **(F)** *Cucumis melo* heat shock cognate 70 kDa protein 2-like (LOC103498170) gene expression.

According to the qRT-PCR results, all of the candidate genes showed higher expression in ms plants than in HM1-1 plants, with the exception that *C. melo* transcription factor *ABORTED MICROSPORES* (*AMS*) (LOC103498166) exhibited a higher expression level in HM1-1 compared with ms-5 plants. Comparing different bud development stages showed higher expression levels of geranylgeranyl transferase type-1 subunit beta (LOC103498167), uncharacterized LOC103498168 (LOC1034981), serine/threonine-protein phosphatase 7-like (LOC103498169) and heat shock cognate 70 kDa protein 2-like (LOC103498170) at the 2-mm bud stage than at the 5-mm bud stage in ms-5 plants, indicating that the expression of these genes increases with the flower development stage. However, for the transcription factor *AMS* (LOC103498166) and RING-H2 finger protein ATL52-like (LOC103498194) genes, the expression levels in 2-mm buds were lower than those in 5-mm buds in ms-5 plants. Compared with HM1-1 (2 mm), the *AMS* gene expression level increased in HM1-1 (5 mm), but in ms-5 (2 mm) and ms-5 (5 mm), *AMS* expression decreased significantly (*P* < 0.01). For the genes *LOC103498166* and *LOC103498167*, expression levels decreased in HM1-1 (5 mm) and ms-5 (5 mm), but the levels significantly increased in ms-5 (2 mm). Compared with HM1-1 (2 mm), the *LOC103498169* gene expression decreased significantly in HM1-1 (5 mm) and ms-5 (5 mm) but increased in ms-5 (2 mm). The *LOC103498170* gene expression level increased significantly in HM1-1 (5 mm) and ms-5 (2 mm) but decreased in ms-5 (2 mm). In contrast, the *LOC103498194* gene expression level decreased significantly in HM1-1 (5 mm) but increased in ms-5 (2 mm) and (5 mm) (**Figure 4**).

**FIGURE 5:**
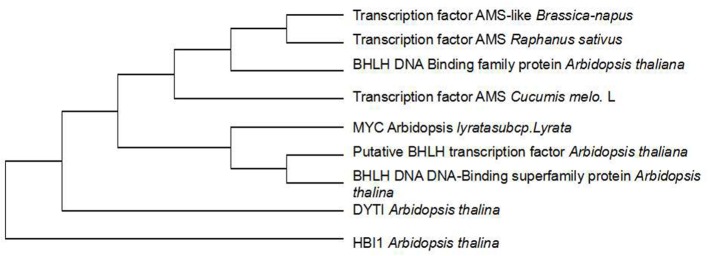
Cluster analysis of candidate and other genes at the *AMS* locus. The transcription factor *AMS Cucumis melo*. L is the candidate gene.

To investigate whether genetic variation of *AMS* is present between ms-5 and HM1-1, the DNA sequence of the *AMS* gene was retrieved from the parental resequencing data and aligned to the DHL92 muskmelon reference genome sequence. The gene structure annotation of *AMS* indicated that this gene had four exons and three introns. The gene alignment of the DNA sequence of *AMS* from ms-5, HM1-1 and the DHL92 reference was analyzed using DNAMAN6.0 software, and multiple alignment methods was used. 85.67% consensus sequence was acquired. We retrieved totally 5 kb sequence may include all promoter region of parental line, Several SNPs were found between ms-5 and HM1-1, alignment of the *AMS* sequences from the parent lines revealed a 8-bp insertion in the promoter regions of ms-5.To investigate whether the mutation in *AMS* is present in DHL92 melon reference lines, we further aligned the DNA sequences from ms-5, HM1-1, and DHL92. Alignment of the nucleotide sequences also revealed a 8-bp insertion mutation in the promoters of ms-5 (**Supplementary Figure S1**). The “G” to “T”mutation type occurred in the male sterility line (ms-5). The result indicated that the loss of *AMS* promoter region may results the male fertility phenotype in melon. To deduce the *AMS* gene function in male sterility traits, multiple DNA sequence alignments of the candidate gene *AMS* and other *BHLH* factor genes were conducted (**Figure 5**). The results indicated that *AMS* has a close evolutionary relationship with transcription factor *ABORTED MICROSPORES-like* protein (*B. napus* and *Raphanus sativus*) and with the basic helix-loop-helix (bHLH) DNA-binding superfamily protein (*Arabidopsis thaliana*). Moderate evolution has occurred between the putative bHLH transcription factor (*A. thaliana*), basic helix-loop-helix (bHLH) DNA-binding superfamily protein (*A. thaliana*), and transcription factor ABORTED MICROSPORES-like (*Camelina sativa*). A short evolutionary distance exists between the DYT1 (*A. thaliana*) and HBI1 (*A. thaliana*) genes (Zhang et al., 2006).

**FIGURE 6:**
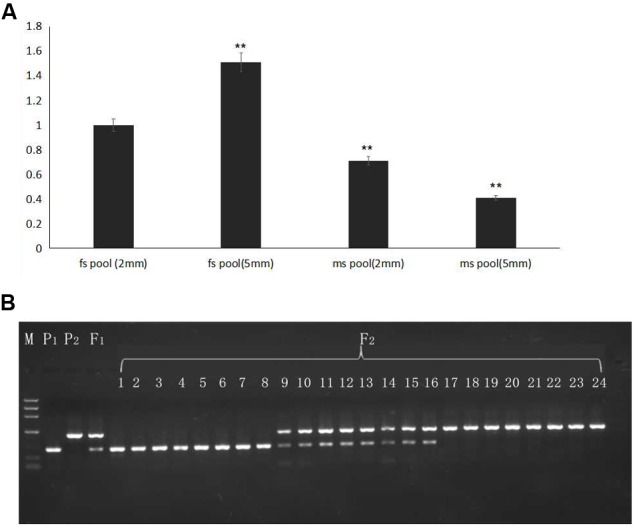
Gene expression verified in F_2_ plants and BSA16 marker amplification in different generations. **(A)** *AMS* gene expression in F_2_ male-sterile and -fertile pools. **(B)** Lined marker amplified in F_2_ homozygous male-sterile and male-fertile plants, and heterozygous F_2_ male-fertile plants. P_1_: ms5; P_2_: HM1-1; F_1_: F_1_ plants from the cross of ms5 and HM1-1; 1–8: male-sterile plants of the F_2_ population; 9–16: male-fertile heterozygous plants in the F_2_ population; 17–24: male-sterile homozygous plants in the F_2_ population. M: the reference markers. ^∗∗^ means significant gene expression level based on fs pool (2 mm).

To detect the expression level of the candidate gene *AMS*, flower buds in 2- and 5-mm stages from pools of 20 homozygous male sterile and 20 fertile F_2_ plants were mixed, and RNA was extracted for qRT-PCR. Selected gene expression in 2-mm buds of the F_2_ male-fertile pool was used as the reference. The results indicated higher expression of the *AMS* gene in 5-mm buds than in 2-mm buds of the F_2_ male-fertile pool; gene expression was significantly lower in the F_2_ male-sterile pool compared with male-fertile pool. Although significant expression was detected in the 2- and 5-mm buds in the male-sterile F_2_ pool, expression levels were lower than those in the 2-mm male-fertile F_2_ pool (**Figure 6A**).

The CAPS marker BSA16 (forward primer TCACTCTTCCTCTTCTCCTTCTCCA, reverse primer TCTCCTCACCACGCCCAATCA) was found linked to the male sterility traits. The recombination of BSA16 and traits determination was 0.16%, 252 F_2_ plants were used to validate the CAPS markers amplification and enzyme digest; the results are shown in **Figure 6B**. The same length of male sterile and fertile in 502 bp, after enzyme digest a fragment of 502 bp was shown in ms-5, and a fragment of 495 bp was shown in HM1-1; both of these fragments were shown in the F_1_ samples. Among the 252 F_2_ plants, 58 plants showed male sterility and 194 plants showed male fertility. The inoculation and molecular marker identification results of F_2_ plants showed a consensus.

## Discussion

The ms-5 line is male sterile, with no pollen in the male flowers. Microscopic observation indicated pollen production capability, but pollination rates of ms-5 were 0 (data not shown). The male fertility is easily observed in the greenhouse. The genetic inheritance analysis of ms-5 traits indicated that its male sterility is controlled by a recessive nucleolar gene. The results were same as those of previous studies (Lecouviour et al., 1990; Pitrat, 1991). To detect whether the ms-5 gene responds to different environmental conditions, ms-5 plants were planted in three places and two different years, which showed that ms-5 is a stable sterile line in multiple conditions. The SLAF-seq method and genome sequences provide significant, useful information for precise gene mapping. In the present study, we first used SLAF-seq technology and combined BSA to detect polymorphic markers between male-sterile and fertile pools. This method provided rapid and precise results in mapping the male sterility gene. Usually, we mapping a candidate gene based on map construction, then found the linked markers to the target traits. But in our study, we first used SFAL-seq method to found the association region, the results gave us the target association region on chromosome 9, we only need to narrow the candidate region in chromosome 9 and add more markers in this region, it is rapid and significant useful method for gene mapping. Four association regions totaling 4.29 Mb in size were obtained in the F_2_ bulk analysis, after which CAPS makers based on parental resequencing and enzyme digest were used to minimize the candidate region. Thirty-two kilobytes with six total candidate genes were ultimately identified and annotated. Based on the inheritance and genetic mapping, the *ms-5* gene was located on chromosome 9. Pitrat (2002) indicated *ms-5* and other male sterility genes were non-allelic; our results confirmed that *ms-5* and *ms-3* were located in different linkage groups of the melon classical genetic map (Park et al., 2004). Several molecular markers were linked to male sterility. Based on the genetic map, there is a long distance between markers BSA1-6 and BSA10. We attempted to minimize this region by adding more markers, but a lengthy empty region was still found, the depth and coverage of genome sequencing could influence these results. BSA16 is a tightly links to the male sterility traits at the genetic distance of 0.2 cM. This marker was tested in F_2_ plants that showed an expected genotype. All selected F_2_ plants in the test gave consistent results, showing that the BSA16 marker is sufficient for MAS breeding work (**Figure 6B**). This marker was designed based on the SNP in LOC103498166, resulting in the products of ms-5 and HM1-1 having different sizes. Using single recessive nuclear male sterility germplasm could reduce the cost of F_1_ seed production in melon, but the heterozygous fertile plants should be removed before pollination. The results of this study on MAS (marker-assisted selection) of male sterility can help identify male sterility and fertility in homozygous and heterozygous plants at seeding. Such an application would reduce the cost of seed production and improve the use of male-sterile lines in melon (McCreight, 1983; McCreight and Elmstrom, 1984; Park et al., 2004; Jin et al., 2016). The present research indicates that the combination of F_2_ bulk SLAF-seq and parental sequencing is a good choice for gene mapping and target gene screening.

The region delimited by the CAPS markers BSA3-3 and BSA16 in the muskmelon DHL92 reference genome was predicted to contain 6 genes (Supplementary Table S2). Among these six candidate genes, compared with previous research, ABORTED MICROSPORES (*AMS*) was indicated to be a candidate gene related to male sterility traits in the target region (Sanders et al., 1999; Thorstensen et al., 2008; Xu et al., 2010; Feng et al., 2012; Xu et al., 2014). *AMS* encodes a basic helix-loop-helix (bHLH) transcription factor that is required for tapetal cell development and postmeiotic microspore formation. The bHLH proteins are part of a superfamily of transcription factors that bind as dimers to specific DNA target sites, and these proteins have been well characterized in non-plant eukaryotes as important regulatory components in diverse biological processes. The AMS transcription factor has been shown to affect the expression of genes involved in several biological pathways (Sorensen et al., 2003; Xu et al., 2010), and, recently, reports of an *AMS* mutant displaying abnormally enlarged tapetal cells and aborted microspore development (Xu et al., 2010) was published. More recently, Xu et al. (2014) indicated that AMS plays a role in cell wall biosynthesis and showed that AMS acts as a principal coordinator of pollen wall formation by directly regulating target genes (Xu et al., 2014). Previous research has shown that the AMS transcription factor is related to male sterility, especially regarding stamen development and function (Thorstensen et al., 2008); is a key transcriptional regulator in pollen wall patterning (Xu et al., 2014); and is a key regulator of both anther development and stamen filament length (Sorensen et al., 2003). In our study, *AMS* was a candidate gene for the ms-5 male sterility. Although five SNPs were found in the *AMS* gene in two parental lines, there was no evidence to support male sterility due to differences in the structure of the *AMS* gene between ms-5 and HM1-1. Compared with the DHL92 reference genome sequence database, all SNPs were located in the exons, but no reference database for the whole gene sequence exists.

The aforementioned SNPs are not unique. The SNPs in ms-5 and the DHL92 reference genomes were the same, but they differed from those of HM1-1. SNP differences between parental lines may due to the ecological type and the sequencing depth (ms-5 and the DHL92 reference genome both belong to cantaloupe, but HM1-1 is a muskmelon type), but not for the male sterility trait. We cannot conclude that these SNPs are related to male sterility. Alignment of nucleotide sequence of *AMS* promoter region from ms-5 male sterile line and male fertile lines (HM1-1 and DHL92) showed that two different mutation types (8-bp deletion, the “G” to “T” mutation) may result in the special function of the gene (**Supplementary Figure S1**). Interestingly, a short 8-bp deletion was found in male fertile lines strongly suggesting that a specific mechanism might underlie this mutation. To answer this question, cloning of the *AMS* transcription factor gene and promoter region sequence analysis should be conducted in the future. However, compared with previous results in *Arabidopsis*, as a regulator, *AMS* control gene expression was not directly involved in the regulation of the target genes (Xu et al., 2010).

For qRT-PCR analysis, we selected two stages (2 and 5 mm length of buds) as the test material based on our previous results of scanning electron microscopy (SEM) observation, which indicated that male-sterile plants had abnormal bud formation at the 2-mm length stage, and significant differences were detected during the tetrad period (2 mm) regarding pollen development. In our study, the *AMS* gene expression level in male-fertile (HM1-1) plants was higher than that in male-sterile (ms-5) plants and the expression level of the AMS gene in ms-5 (2 mm) was significantly lower than that in ms-5 (5 mm); qRT-PCR results indicated that the *AMS* gene expression level was different from that of other genes. With the exception of the AMS gene, the five other candidate genes showed high expression levels in male-sterile plants but low expression levels in fertile plants. We assumed that the *AMS* gene would express higher levels in fertile plants, but this was not the case. Previous research indicated that gene expression levels related to male sterility could be higher or lower. These genes play a role as a positive or negative regulator (Zhang et al., 2006; Feng et al., 2012; Ma et al., 2012; Gu et al., 2014; Figueroa and Browse, 2015). The present results were similar to those of Xu et al. (2010), who indicated that the ABORTED MICROSPORES (AMS) mutant exhibits abnormal retardation of degeneration of the tapetum as well as collapse of microspores, which is a positive regulator for tapetum PCD. In *A. thaliana*, male sterile mutant AMS (ABORTED MICROSPORES) exhibits abnormally degeneration retardation of the tapetum as well as collapse of microspores was reported (Xu et al., 2010). To better understand the regulatory role of *AMS* during anther development in melon male sterility and fertility buds, SEM and TEM (transmission electron microscopy) observation will be conducted for identify abnormal formation of pollen wall in further. And the further work aiming to valid the discovered male sterility haplotype, AMS transfactor will be transfered to *A. thaliana AMS* mutant, and melon male sterility germplasm. Transcript sequencing analysis between male sterility and male fertility buds is also conducting for valid the gene function of under the regulated by AMS.

In our study, BLAST analysis and cluster analysis of candidate genes with other functional genes indicated that *AMS* shows homology to other published *Arabidopsis* sequences (more than 90% homology) (Sorensen et al., 2003). This close sequence homology of the *AMS* transcription factor was found for *Brassica* and *Raphanus* as well as the BHLH sequence from *Arabidopsis*. The *AMS* protein is predicted to belong to the MYC class of BHLH transcription factors and plays an important role in pollen wall formation. In contrast to our findings, *AMS* may regulate melon male sterility indirectly, as occurs in several candidate genes in other species. Further research should be conducted to identify the genetic function of *AMS* transcription factors.

## Author Contributions

YS contributed to the conception and design of the work and created the draft of the manuscript. YW constructed the genetic map for the work. SJ mapped genetic male sterility traits and collected and analyzed all the data. YJ analyzed the qRT-PCR data. PJ interpreted and prepared all the figures and tables; and FL revised the manuscript critically for important intellectual content.

## Conflict of Interest Statement

The authors declare that the research was conducted in the absence of any commercial or financial relationships that could be construed as a potential conflict of interest.

## Footnotes

1 https://melonomics.net/files/Genome/Melon_genome_v3.5.1/

2 http://www.premierbiosoft.com/

3 http://helix.wustl.edu/dcaps/dcaps.html

4 http://blast.ncbi.nlm.nih.gov/Blast.cgi/

5 http://cuke.vcru.wisc.edu/wenglab/

6 http://www.softberry.com/berry.phtml?topic=fgenes_plus&group=programs&subgroup=gfs

7 http://www.softberry.com/berry.phtml?topic=index\&group=programs\&subgroup=gfind

8 https://melonomics.net/files/Genome/Melon_genome_v3.5.1/

9 http://www.softberry.com/berry.phtml?topic=index\&group=programs\&subgroup=gfind

## Supplementary Material

Figure S1 Alignment of DNA sequences of the *AMS* gene from Male sterile lines (ms-5) and male fertile line (HM1-1 and DHL92). The two types of mutations are shaded in yellow and red. Multiple sequence alignment was done by DNAMAN software6.0.
