# Supplementary material for: Mapping and Preliminary Analysis of ABORTED MICROSPORES (AMS) as the Candidate Gene Underlying the Male Sterility (MS-5) Mutant in Melon (Cucumis melo L.)
Source: Front Plant Sci. 2017 May 30;8:902. doi: 10.3389/fpls.2017.00902 (PMC5447745; doi:10.3389/fpls.2017.00902)
Supplement: Figure S1 — Alignment of DNA sequences of the AMS gene from Male sterile lines (ms-5) and male fertile line (HM1-1 and DHL92). The two types of mutations are shaded in yellow and red. Multiple sequence alignment was done by DNAMAN software6.0. [file Image_1.PDF]

Fig.S1

```
ms-5        ACTTGCGACGATGAAATTGGGCTCGGTAAGGTGGATCAACACGAAACTTCGGCTAGGGTT
HM1-1       ACTTGCGACGATGAAATTGGGCTCGGTAAGGTGGATCAACACGAAACTTCGGCTAGGGTT
DHL92       ACTTGCGACGATGAAATTGGGCTCGGTAAGGTGGATCAACACGAAACTTCGGCTAGGGTT
            ************************************************************

ms-5        GACGACAAGGTCTGATGGCGGTGGAGGCTCGTCGACGGCGGAACAGACAGCGAGTCTTGG
HM1-1       GACGACAAGGTCTGATGGCGGTGGAGGCTCGTCGACGGCGGAATAGACGGCGAGTCTTGG
DHL92       GACGACAAGGTCTGATGGCGGTGGAGGCTCGTCGACGGCGGAACAGACAGCGAGTCTTGG
            ******************************************* **** ***********

ms-5        TTGAAGTTTGAGAGGTTAGGGTTTCTTGAGGAAGGAGAAGATGAATAGTAACCTCACGCA
HM1-1       TTGAAGTTTGAGAGGTTAGGGTTTCTTGAGGAAGGAGAAGATGAATAGTAACCTCACGCA
DHL92       TTGAAGTTTGAGAGGTTAGGGTTTCTTGAGGAAGGAGAAGATGAATAGTAACCTCACGCA
            ************************************************************

ms-5        TCACGAGGTACTATTTAAAGAAATAATATTTTTATTTTTATTTTTTACTTTTACTTTTAC
HM1-1       TCACGAGGTCCTATTTAAAGAAATAATATTTTTATTTTTTTATTTTTTTTTTACTTTTAC
DHL92       TCACGAGGTACTATTTAAAGAAATAATATTTTTATTTTTATTTTTTACTTTTACTTTTAC
            ********* ***************************** * ****  ************

ms-5        TTTTTCCTTCTTTTCTCAATCTTTTCCAACTAAACCAAATAAATCTTCTCTTTCTTTGTT
HM1-1       TTTTTCCTTCTTTTCTCAATCTTTTCCAACTAAACCAAATAAATCTTCTCTTTCTTTGTT
DHL92       TTTTTCCTTCTTTTCTCAATCTTTTCCAACTAAACCAAATAAATCTTCTCTTTCTTTGTT
            ************************************************************

ms-5        AAATACCAACCAAAACCCCTTTTTAAACTCAAATACCTCTCTCCTCAATAACTCTTCTCA
HM1-1       AAATACCAACCAAAACCCCTTTTTAAACTCAAATACCTCTCTCCTCAATAACTCTTCCCA
DHL92       AAATACCAACCAAAACCCCTTTTTAAACTCAAATACCTCTCTCCTCAATAACTCTTCTCA
            ********************************************************* **

ms-5        TCAAATCTTCTTTTTATCTTCCCAAAACAATATATATTCTTAAATAATTATATTATCTTC
HM1-1       TCAAATCTTCTTTTTATCTTCCCAAAACAATATATATTCTTAAATAATTATATTATCTTC
DHL92       TCAAATCTTCTTTTTATCTTCCCAAAACAATATATATTCTTAAATAATTATATTATCTTC
            ************************************************************

ms-5        ATAATATAATTATTTTAACTTTAAATTCAATTAATTATATAAAATTATATAATCATATAC
HM1-1       ATAATATAATTATTTTAACTTTAAATTCAATTAATTATATAANNNNNNNNAATCATATAT
DHL92       ATAATATAATTATTTTAACTTTAAATTCAATTAATTATATAANNNNNNNNAATCATATAC
            ******************************************        ********* 

ms-5        ATAATTAATCATAATTCCCTCGATACAATTCTCTCAACCACAATTCACCTAAAACAACCA
HM1-1       ATAATTAATCATAATTCCCTCAATACAATTCTCTCAACCACAATTCACCTAAAACAACCA
DHL92       ATAATTAATCATAATTCCCTCGATACAATTCTCTCAACCACAATTCACCTAAAACAACCA
            ******************** ***************************************
```

```
ms-5     ATTTTAATCATAAATTCCAAATTTAAAAATAATTAATTAGATATTTTTCTAAAATATCTA
HM1-1    ATTTTAATCATAAATTCCAAATTTAAAAATAATTAATTAGATATTTTTCTAAAATATCTA
DHL92    ATTTTAATCATAAATTCCAAATTTAAAAATAATTAATTAGATATTTTTCTAAAATATCTA
         ************************************************************

ms-5     ATTAATTCTAATTCCTACAAAATCAATAATTTCAAAAATTAAATCAAATAACACCCAAAA
HM1-1    ATTAATTCTAATTCCTACAAAATCAATAATTTCAAAAATTAAATCAAATAACACCCAAAA
DHL92    ATTAATTCTAATTCCTACAAAATCAATAATTTCAAAAATTAAATCAAATAACACCCAAAA
         ************************************************************

ms-5     TAATTTCGAAATTAGTCAAAATTAAACTTATAATTAAGATTTAAAGTTACCTTAATTTGG
HM1-1    TAATTTCAAAATTAGTCAAAATTAAACTTATAATTAAGATTTAAAGTTACCTTAATTTGG
DHL92    TAATTTCGAAATTAGTCAAAATTAAACTTATAATTAAGATTTAAAGTTACCTTAATTTGG
         ******* ****************************************************

ms-5     GGTGTTACACTTTTAGTTTAAATTGGTGATTTAAGACTACCAAAGATACGATGAAATTTT
HM1-1    GGTGTTACACTTTTAGTTTAAATTGGTGATTTAAGACTACCAAAGATACGATGAAATTTT
DHL92    GGTGTTACACTTTTAGTTTAAATTGGTGATTTAAGACTACCAAAGATACGATGAAATTTT
         ************************************************************

ms-5     ATCTTTAATTATTCACCCCAACGTGTTGTTTCATCATCCAATTCAAATAGACTAATAGCA
HM1-1    ATCTTTAATTATTCACCCCAACGTGTTGTTTCATCATCCAATTCAAATAGACTAATAGCA
DHL92    ATCTTTAATTATTCACCCCAACGTGTTGTTTCATCATCCAATTCAAATAGACTAATAGCA
         ************************************************************

ms-5     TTTGAAACAAATATCAAAATTTAAAAATATCTTAAAATAATAATAAGGCAAAATACCTTT
HM1-1    TTTGAAACAAATATCAAAATTTAAAAATATCTTAAAATAATAATAAGGAAAAATACCTTT
DHL92    TTTGAAACAAATATCAAAATTTAAAAATATCTTAAAATAATAATAAGGCAAAATACCTTT
         ************************************************ ***********

ms-5     TTAGTCCCTAAAATTTGGGGTTAGTGTTTCTCTAGTCCATGAATTCAAAAGGAAAACTTT
HM1-1    TTAGTCCCTAAAATTTGGGGTTAGTGTTTCTCTAGTTCATGAATTCAAAAGGAAAACTTT
DHL92    TTAGTCCCTAAAATTTGGGGTTAGTGTTTCTCTAGTCCATGAATTCAAAAGGAAAACTTT
         ************************************ ***********************

ms-5     TTTTCCTATGTAAAGAGCAATCTAAACAAAGTTTAAGCTCAATGAAAAAAAGTCTCATTT
HM1-1    TTTTCCTATGTAAAGAGCAATCTAAACAAAGTTTAAGCTCAATGAAAAAAAGTCTCATTT
DHL92    TTTTCCTATGTAAAGAGCAATCTAAACAAAGTTTAAGCTCAATGAAAAAAAGTCTCATTT
         ************************************************************

ms-5     TGATTTATTTACTTTTAAAACGTGTATTTCATATACCTTTAGGTTCATATACTTACGTTT
HM1-1    TGATTTATTTACTTTTAAAACGTGTATTTCATATACCTTTAGGTTCATATACTTACGTTT
DHL92    TGATTTATTTACTTTTAAAACGTGTATTTCATATACCTTTAGGTTCATATACTTACGTTT
         ************************************************************
```

```
ms-5      CTTCAAAGTACTTTTGGTTATTTTGGTTTATTTTTCTTCTTATATTTCATCTTTTTCGAC
HM1-1     CTTCAAAGTACTTTTGGTTATTTTGGTTTATTTTTCTTCTTATATTTCATCTTTTTCGAC
DHL92     CTTCAAAGTACTTTTGGTTATTTTGGTTTATTTTTCTTCTTATATTTCATCTTTTTCGAC
          ************************************************************

ms-5      CACAATTTTAAAATAACAGAACACTCGACGAATCTCTCAATCCATAATTCTGTAGTAAAG
HM1-1     CACAATTTTAAAATAACAGAACACTCGACAAATCTCTCAATCCATAATTCTGTAGTAAAG
DHL92     CACAATTTTAAAATAACAGAACACTCGACGAATCTCTCAATCCATAATTCTGTAGTAAAG
          ***************************** ******************************

ms-5      AGCTTGCGTTGTATAAAGAAATTTTGTCATGTTTTTGAACATAAAAGACCAAAACATTTT
HM1-1     AGCTTGCGTTGTATAAAGAAATTTTGTCATGTTTTTGAACATAAAAGACCAAAACATTNT
DHL92     AGCTTGCGTTGTATAAAGAAATTTTGTCATGTTTTTGAACATAAAAGACCAAAACATTTT
          ********************************************************** *

ms-5      TTTTAAAAAAAAAGTTCATAATCTAAAAAATACATTTTAAAATACATGAAATAAAAAGAG
HM1-1     TTTAAAAAAAAAAGTTCATAATCTAAAAAATACATTTTAAAATACATGAAATAAAAAGAG
DHL92     TTTTAAAAAAAAAGTTCATAATCTAAAAAATACATTTTAAAATACATGAAATAAAAAGAG
          *** ********************************************************

ms-5      AAAGACAGAGAAAGTAGACAAACTAAATCAATGTTTCGAACAATTTATAAATTAGACTAA
HM1-1     AAAGATAGAGAAAGTAGACAAACTAAATCAATGTTTCGAACAATTTATAAATTAGACTAA
DHL92     AAAGACAGAGAAAGTAGACAAACTAAATCAATGTTTCGAACAATTTATAAATTAGACTAA
          ***** ******************************************************

ms-5      TTCGTTCAAATTATTATAAAAGCTATAAATAAAAAATTAAATAGTTACCAAACAAAGTCA
HM1-1     TTCGTACAAATTATTATAAAAACTATAAATAAAAAATTAAATAGTTACCAAACAAAGTCA
DHL92     TTCGTTCAAATTATTATAAAAGCTATAAATAAAAAATTAAATAGTTACCAAACAAAGTCA
          ***** *************** **************************************

ms-5      AAACTAATACCAAACAGTGATGCGACAATAATCAATCACATATAGAATCTCAATCAAAAG
HM1-1     AAACTAATACCAAACAGTGATGCGACAATAATCAATCACTTATAGAATCTCAATCAAAAG
DHL92     AAACTAATACCAAACAGTGATGCGACAATAATCAATCACATATAGAATCTCAATCAAAAG
          *************************************** ********************

ms-5      AAATAAGAATATATGTGACAACATGTGTGGCTACTTTGATATATAAAGCATGTAGATGTA
HM1-1     AAATAAGAATATATGTGACAACATGTGTGGCTACTTTGATATATAAAGCATGTAGATGTA
DHL92     AAATAAGAATATATGTGACAACATGTGTGGCTACTTTGATATATAAAGCATGTAGATGTA
          ************************************************************

ms-5      AGCATGTTTGTAAGTACCTTAAATTGTTTGGATATATATACAATTAACACAATCATCACA
HM1-1     AGCATGTTTGTAAGTACCTTAAATTGTTTGGATATATATACAATTAACACAATCATCACA
DHL92     AGCATGTTTGTAAGTACCTTAAATTGTTTGGATATATATACAATTAACACAATCATCACA
          ************************************************************

ms-5      TCATGACATATTAAGACATTCAGTTCTATGTTAAGAGTAGTTGAAGTCCTGAAAGAAGAT
HM1-1     TCATGACATATTAAGACATTCAGTTCTATGTTAAGAGTAGTTGAAGTCCTGAAAGAAGAT
```

```
DHL92          TCATGACATATTAAGACATTCAGTTCTATGTTAAGAGTAGTTGAAGTCCTGAAAGAAGAT
               ************************************************************

ms-5           GCAAAAAAAAAAAAAAAGCACTTTTCATTTCTGAATTTTTAGAAAAGTAGGGTAACAATTT
HM1-1          GCAAAAAAAAAAAAAAAGCACTTTTCATTTCTGAATTTTTAGAAAAGTAGGGTAACAATTT
DHL92          GCAAAAAAAAAAAAAAAGCACTTTTCATTTCTGAATTTTTAGAAAAGTAGGGTAACAATTT
               ************************************************************

ms-5           AGTTCATGTAGTTTTATTTTACTGATGTAACAATTTACTTTTAATTTTGTAACAATTTAG
HM1-1          AGTTCATGTAGTTTTATTTTACTGATGTAACAATTTACTTTTAATTTTGTAACAATTTAG
DHL92          AGTTCATGTAGTTTTATTTTACTGATGTAACAATTTACTTTTAATTTTGTAACAATTTAG
               ************************************************************

ms-5           TATATAAATTAATTTAGCATACGACAATTTAGTCACTGACGTAATAAAAAAGAATTCATC
HM1-1          TATATAAATTAATTTAGCATACGACAATTTAATNNNCAATTTAATAAAAAGGAATTCATC
DHL92          TATATAAATTAATTTAGCATACGACAATTTAGTCACTGACGTAATAAAAAAGAATTCATC
               ****************************** *     *  ********* **********

ms-5           AAAATATTGTATTATGTAAAAATAGTGAGCCCTATGATTGGTTTATTTACGTAAAAATCT
HM1-1          AAAATATTGTATTATGTAAAAATAGTGAGCCCTATGATTGGTTTATTTACGTAAAAATCT
DHL92          AAAATATTGTATTATGTAAAAATAGTGAGCCCTATGATTGGTTTATTTACGTAAAAATCT
               ************************************************************

ms-5           AATTAAACCTTAACACTAATGTCTAGGAAGACTGAATGTTATATTGTTATATATAATTTA
HM1-1          AATTAAACCTTAACACTAATGTCTAGGAAGATTGAATGTTATATTGTTATATATAATTCA
DHL92          AATTAAACCTTAACACTAATGTCTAGGAAGACTGAATGTTATATTGTTATATATAATTTA
               ******************************* ************************** *

ms-5           AAAACTAAATAGTTATATAAAATTGAAAATACAGAAAATAAATCATTACACAGAAAAGGG
HM1-1          AAAACTAAATAGTTATATAAAATTGAAAATACAGAAAATAAATCATTACACAGGAAAGGG
DHL92          AAAACTAAATAGTTATATAAAATTGAAAATACAGAAAATAAATCATTACACAGAAAAGGG
               ***************************************************** ******

ms-5           AAGGGAAGTTGAGGAGTAAATTGTCAGTTTAGTTTAGGGAGTACGAATAAATTTTAATGT
HM1-1          AAGGGAAGTTGAGGAGTAAATTGTCAGTTTAGTTTAGGGAGTACGAATAAATTTTAATGT
DHL92          AAGGGAAGTTGAGGAGTAAATTGTCAGTTTAGTTTAGGGAGTACGAATAAATTTTAATGT
               ************************************************************

ms-5           AGAATTAAAATAAACTTAGCAAGGCAGTTAGGGTATCTTAGGTCATGCAATCTGTTCCTC
HM1-1          AGAATTAAAATAAACTTAGCAAGGCAGTTAGGGTATCTTAGGTCATGCAATCTGCTCCTC
DHL92          AGAATTAAAATAAACTTAGCAAGGCAGTTAGGGTATCTTAGGTCATGCAATCTGTTCCTC
               ****************************************************** *****

ms-5           CTACCAACTCATCTACATGTTTCTTCCTTTAATTTATAAAAAGAAATATATCCTAAAAAG
HM1-1          CTACCAACTCATCTACATGTTTCTTCCTTTAATTTATAAAAAGAAATATATCCTAAAAAG
DHL92          CTACCAACTCATCTACATGTTTCTTCCTTTAATTTATAAAAAGAAATATATCCTAAAAAG
               ************************************************************
```

```
ms-5     CCTCTCTATCTCTCTCTCTCTCTCATGCATGGAGTGAAATTCACCTACCAAAATTTGAAC
HM1-1    CCTCTCTATCTCTCTCTCTCTCTCATGCATGGAGTGAAATTCACCTACCAAAATTTGAAC
DHL92    CCTCTCTATCTCTCTCTCTCTCTCATGCATGGAGTGAAATTCACCTACCAAAATTTGAAC
         **********************************************************

ms-5     TAAACAACCAAAATTATATAAAAAGAAACAAGTGTAGAACAAAAAGTATAGTACTACTTC
HM1-1    TAAACAACCAAAATTATATAAAAAGAAACAAGTGTAGAACAAAAAGTATAGTACTACTTC
DHL92    TAAACAACCAAAATTATATAAAAAGAAACAAGTGTAGAACAAAAAGTATAGTACTACTTC
         ************************************************************

ms-5     TACTTCAGCTGATCATTGTTTGTATTCATCTATTTCTCTCTTTGTTTCTAAAGAAAAAAA
HM1-1    TACTTCAGCTGATCATTGTTTGTATTCATCTATTTCTCTCTTTGTTTCTAAAGAAAAGAA
DHL92    TACTTCAGCTGATCATTGTTTGTATTCATCTATTTCTCTCTTTGTTTCTAAAGAAAAAAA
         ********************************************************* **

ms-5     AAAAAAAAGGTGGCAATTTGGTTGGCCATTGGTCTGAGTTGGTGGGTTTTGTCTGAAACT
HM1-1    AAAAAAAAGGTGGCAATTTGGTTGGCCATTGGTCTGAGTTGGTGGGTTTTGTCTGAAACT
DHL92    AAAAAAAAGGTGGCAATTTGGTTGGCCATTGGTCTGAGTTGGTGGGTTTTGTCTGAAACT
         ************************************************************

ms-5     TTTGAGGGTTATATGATGCCATTCTCATGGTCCAGTTGTTAACTATTGCTATCAATTGAA
HM1-1    TTTGAGGGTTATATGATGCCATTCTCATGGTCCAGTTGTTAACTATTGCTATCAATTGAA
DHL92    TTTGAGGGTTATATGATGCCATTCTCATGGTCCAGTTGTTAACTATTGCTATCAATTGAA
         ************************************************************

ms-5     GTACTGTTTTATCAAATCTTTTTAACCCTTTGTTTTTTCTTGGAGAGGATCCTGTGCTAT
HM1-1    GTACTGTTCTATCAAATCTTTTTAACCCTTTGTTTTTTCTTGGAGAGGATCCTGTGCTAT
DHL92    GTACTGTTTTATCAAATCTTTTTAACCCTTTGTTTTTTCTTGGAGAGGATCCTGTGCTAT
         ******** ***************************************************

ms-5     TGTCTTCTAGGTTCCGATGTGCCTTTGAGGAAACTGTTTCATTCAAATTTGTACATAATT
HM1-1    TGTCTTCTAGGTTCCGATGTGCCTTTGAGGAAACTGTTTCATTCAAATTTGTACATAATT
DHL92    TGTCTTCTAGGTTCCGATGTGCCTTTGAGGAAACTGTTTCATTCAAATTTGTACATAATT
         ************************************************************

ms-5     CTCTTTGAGTATATGATTCTCTGACCAAATCTATCTATTATTGAATGAGATGTTTCTTTT
HM1-1    CTCTTTGAGTATATGATTCTCTGACCAAATCTATCTATTATTGAATGAGATGTTTCTTTT
DHL92    CTCTTTGAGTATATGATTCTCTGACCAAATCTATCTATTATTGAATGAGATGTTTCTTTT
         ************************************************************

ms-5     TGGTATGTTGAATGTTATAATTTCTAACTATTCTCTACTTTTTCTAATAAAAAATGCAGG
HM1-1    TGGTATGTTGAATGTTATAATTTCTAATTATTCTCTACTTTTTCTAATAAAAAATGCAGG
DHL92    TGGTATGTTGAATGTTATAATTTCTAACTATTCTCTACTTTTTCTAATAAAAAATGCAGG
         *************************** ********************************

ms-5     CGATTCTAAACATAGCCATTGTCATCATGCAAATAAACCTTGTGGAGAGACTAAGACCCC
```

```
HM1-1          CGATTCTAATCATAGCCATTGTCATCATGCAAATAAACCTTGTGGAGAGACTAAGACCCC
DHL92          CGATTCTAAACATAGCCATTGTCATCATGCAAATAAACCTTGTGGAGAGACTAAGACCCC
               ********* **************************************************

ms-5           TTGTGGGTACTAAAAGTTGGGATTATTGTGTTCTTTGGAAATTGAGCCAAGACCAAAGGT
HM1-1          TTGTGGGTACTAAAAGTTGGGATTATTGTGTTCTTTGGAAATTGAGCCAAGACCAAAGGT
DHL92          TTGTGGGTACTAAAAGTTGGGATTATTGTGTTCTTTGGAAATTGAGCCAAGACCAAAGGT
               ************************************************************

ms-5           TAGGTTGAGTTACTACTTTATTCTCTCTCTCATTCCTAATCTTATTTCTCTCTTTTTCCT
HM1-1          TAGGTTGAGTTACTACTTTATTCTCTCTCTCATTCCTAATCTTATTTCTCTCTTTTTCCT
DHL92          TAGGTTGAGTTACTACTTTATTCTCTCTCTCATTCCTAATCTTATTTCTCTCTTTTTCCT
               ************************************************************

ms-5           TTTTTTTTTTTTTTCATGTTTTCAGATGTATTGAATGGATGGATTGTTGTTGCGCTGGGA
HM1-1          TTTTTTTTTTTTTTCATGTTTTCAGATGTATTGAATGGATGGATTGTTGTTGCGCTGGGA
DHL92          TTTTTTTTTTTTTTCATGTTTTCAGATGTATTGAATGGATGGATTGTTGTTGCGCTGGGA
               ************************************************************

ms-5           CTGAGAACAATAATCAAAATGGTAGTGGAGAAGAAGAACTTGTACTTCCTGTTTCTCAAG
HM1-1          CTGAGAACAATAATCAAAATGGTAGTGGAGAAGAAGAACTTGTACTTCCTGTTTCTCAAG
DHL92          CTGAGAACAATAATCAAAATGGTAGTGGAGAAGAAGAACTTGTACTTCCTGTTTCTCAAG
               ************************************************************

ms-5           TCATTCAATGTAGGGACACTATNTGTCCACACCCAAGAGCTAGTTCTTGTGAACTTCTTG
HM1-1          TCATTCAATGTAGGGACACTATATGTCCACACCCAAGAGCTAGTTCTTGTGAACTTCTTG
DHL92          TCATTCAATGTAGGGACACTATATGTCCACACCCAAGAGCTAGTTCTTGTGAACTTCTTG
               ********************** *************************************

ms-5           ATCAGTTGCCATGTTCAATGCCACTAAATTCTGGGTATTCCATTCACACTTATCATCTCC
HM1-1          ATCAGTTGCCATGTTCAATGCCACTAAATTCTGGGTATTCCATTCACACTTATCATCTCC
DHL92          ATCAGTTGCCATGTTCAATGCCACTAAATTCTGGGTATTCCATTCACACTTATCATCTCC
               ************************************************************

ms-5           ACATACCCAATCTCTTTGATCTCTCAGATGTTCTCTGTTGAACAGGGTTTATATACAAAC
HM1-1          ACATACCCAATCTCTTTGATCTCTCAGATGTTCTCTGTTGAACAGGGTTTATATACAAAC
DHL92          ACATACCCAATCTCTTTGATCTCTCAGATGTTCTCTGTTGAACAGGGTTTATATACAAAC
               ************************************************************

ms-5           ATTGCTAACAAATGAACCCAACTGGCTACTCTTCTCCAACGCCGCCGATTCCACCGTCCT
HM1-1          ATTGCTAACAAATGAACCCAACTGGCTACTCTTCTCCAACGCCGCCGATTCCACCGTCCT
DHL92          ATTGCTAACAAATGAACCCAACTGGCTACTCTTCTCCAACGCCGCCGATTCCACCGTCCT
               ************************************************************

ms-5           GGACGTGAGTAGCATTTGCAAGGAACATTACCATATATTACTACATACATGTCATTGCAA
HM1-1          GGACGTGAGTAGCATTTGCAAGGAACATCACCATATATTACTACATACATGTCATTGCAA
DHL92          GGACGTGAGTAGCATTTGCAAGGAACATTACCATATATTACTACATACATGTCATTGCAA
```

```
              **************************** *******************************

ms-5          CAAAAAGTTACACTTTTTTTCTTCATCTCTTTGCAGGAAACAATGGTGACCAGAGTATTA
HM1-1         CAAAAAGTTACACTTTTTTTCTTCATCTCTTTGCAGGAAACAATGGTGACCAGAGTATTA
DHL92         CAAAAAGTTACACTTTTTTTCTTCATCTCTTTGCAGGAAACAATGGTGACCAGAGTATTA
              ************************************************************

ms-5          GTTCCATTTGCATTTGGATTAGTGGAGTTGTTCGCTGCTAAACATGTATAGACACACACT
HM1-1         GTTCCATTTGCATTTGGATTAGTGGAGTTGTTCGCTGCTAAACATGTATAGACACACACT
DHL92         GTTCCATTTGCATTTGGATTAGTGGAGTTGTTCGCTGCTAAACATGTATAGACACACACT
              ************************************************************

ms-5          TCTAGTAATCTCATCTCTATATACACTATCTAATCACTTAGGCTATCGGGAGAAACATTT
HM1-1         TCTAGTAATCTCATCTCTATATACACTATCTAATCACTTAGGCTATCGGGAGAAACATTT
DHL92         TCTAGTAATCTCATCTCTATATACACTATCTAATCACTTAGGCTATCGGGAGAAACATTT
              ************************************************************

ms-5          GATTTCTCCTTTTTGTACCTTCTAGTGATTAACTTATAAACTTACATAACCTAATCTTAC
HM1-1         GATTTCTCCTTTTTGTACCTTCTAGTGATTAACTTATAAACTTACATAACCTAATCTTAC
DHL92         GATTTCTCCTTTTTGTACCTTCTAGTGATTAACTTATAAACTTACATAACCTAATCTTAC
              ************************************************************

ms-5          ATTAAAACCATTAAATTATTATTAAATTTGTGTAATTGAATATTTGATGTTATTCACATG
HM1-1         ATTAAAACCATTAAATTATTATTAAATTTGTGTAATTGAATATTTGATGTTATTCACATG
DHL92         ATTAAAACCATTAAATTATTATTAAATTTGTGTAATTGAATATTTGATGTTATTCACATG
              ************************************************************

ms-5          GATTTTATTGGTTTAGAAATTTGATTGTCCCTAATATATTTTATTCCATATCAAAATGTG
HM1-1         GATTTTATTGGTTTAGAAATTTGATTGTCCCTAATATATTTTATTCCATATCAAAATCTG
DHL92         GATTTTATTGGTTTAGAAATTTGATTGTCCCTAATATATTTTATTCCATATCAAAATGTG
              ********************************************************* **

ms-5          AATAAACATATTTATAAACCTAAATATATCATTCACGTTTAGATTTTTTTTTCTTTTTTA
HM1-1         AATAAACATATTTATAAACCTAAATATATCATTCACGTTTAGATTTTTTTTTCTTTTTTA
DHL92         AATAAACATATTTATAAACCTAAATATATCATTCACGTTTAGATTTTTTTTTCTTTTTTA
              ************************************************************

ms-5          ATATATACCGAATTTGTGATTTCTTTTTACCAAAATTTTATCATTCACATGAATTTTATT
HM1-1         ATATATACCGAATTTGTGATTTCTTTTTACCAAAATTTTATCATTCACATGAATTTTATT
DHL92         ATATATACCGAATTTGTGATTTCTTTTTACCAAAATTTTATCATTCACATGAATTTTATT
              ************************************************************

ms-5          TCAATTTTGGGTTTTTATTTTATTTTTTGAATCAAAATTGGCTTCTGTATTTTATATTTT
HM1-1         TCAATTTTGGGTTTTTATTTTATTTTTTGAATCAAAATTGGCTTCTGTTTTTTANATTTT
DHL92         TCAATTTTGGGTTTTTATTTTATTTTTTGAATCAAAATTGGCTTCTGTATTTTAAATTTT
              ************************************************ ***** *****
```

```
ms-5      TTTATTTTTTGTAAAAAAATTACCATTCACGATCTTTTATCACAACTTTATTTGGTTTTA
HM1-1     TTTAATTTTTGTAAAAAATTTACCATTCACGATCTTTTATCACAACTTTATTTGGTTTTA
DHL92     TTTATTTTTTGTAAAAAAATTACCATTCACGATCTTTTATCACAACTTTATTTGGTTTTA
          **** ************* *****************************************

ms-5      GTTTTTATTTGATTTTTTTAATCTACTGTTATTATTATTGGGTTGTTTTCTAAAATTTGA
HM1-1     GTTTTTATTTGATTTTTTTAATCTACTGTTATTATTATTGGGTTGTTTTCTAAAATTTGA
DHL92     GTTTTTATTTGATTTTTTTAATCTACTGTTATTATTATTGGGTTGTTTTCTAAAATTTGA
          ************************************************************

ms-5      TTTTTTTTTTTTTTTTTCAATTTTTCTAAGGTTTGATTTAAGATTTAAATCTCATGTAAT
HM1-1     TTTTTTTTTTTTTGTTGCAATTTTTCTAAGGTTTGATTTAAGATTTAAATCTCATGTAAT
DHL92     TTTTTTTTTTTTTGTTTCAATTTTTCTAAGGTTTGATTTAAGATTTAAATCTCATGTAAT
          ************* ** *******************************************

ms-5      TTAATACTAAACTATTAAACAAAATGTTCCCTATCTTGTATGACTTTAACCATAAATGAA
HM1-1     TTAATACTAAACTATTAAACAAAATGTTCCCTATCTTGTATGACTTTAACCATAAATGAA
DHL92     TTAATACTAAACTATTAAACAAAATGTTCCCTATCTTGTATGACTTTAACCATAAATGAA
          ************************************************************

ms-5      TATAAATTTTCAAAACTAAAAATAAATAAACAAAATCTATAATTCATTTTAAAAGAAAAA
HM1-1     TATAAATTTTCAAAACTAAAAATAAATAAACAAAATCTATAATTCATTTTAAAAGAAAAA
DHL92     TATAAATTTTCAAAACTAAAAATAAATAAACAAAATCTATAATTCATTTTAAAAGAAAAA
          ************************************************************

ms-5      CCTCAAAATTTCTAAAATTCACGTTCAAATGCCATTTTTTTCATTTTATTTAAATTGGAA
HM1-1     CCTCAAAATTTCTAAAATTCACGTTCAAATGCCATTTTTTTCATTTTATTTAAATTGGAA
DHL92     CCTCAAAATTTCTAAAATTCACGTTCAAATGCCATTTTTTTCATTTTATTTAAATTGGAA
          ************************************************************

ms-5      ATAAATTAGATTATTCTAAAACCTCACCTCAGAATCACCAACCACAAATCTAAATTTTTT
HM1-1     ATAAATTAGATTATTCTAAAACCTCACCTCAGAATCACGAACCACAAATCTAAATTTTTT
DHL92     ATAAATTAGATTATTCTAAAACCTCACCTCAGAATCACCAACCACAAATCTAAATTTTTT
          ************************************** *********************

ms-5      AAAAATAAATAAATAAAATTAAGATTTCAAATCTAAATTCCCTCTCTCTTTTCTTATCTT
HM1-1     AAAAATAAATAAATAAAATTAAGATTTCAAATCTAAATTTCCTCTCTCTTTTCTTATCTT
DHL92     AAAAATAAATAAATAAAATTAAGATTTCAAATCTAAATTCCCTCTCTCTTTTCTTATCTT
          *************************************** ********************

ms-5      TTCTTTTCATTTTGCTTTTAGTTTTGTTAACTCACTCATCCTATTTTTTAAAAAAAAAAA
HM1-1     TTCTTTTCATTTTGCTTTTAGTTTTGTTAACTCACTCATCCTATTTTTTAAAAAAAAAAA
DHL92     TTCTTTTCATTTTGCTTTTAGTTTTGTTAACTCACTCATCCTATTTTTTAAAAAAAAAAA
          ************************************************************

ms-5      AATCTTTGACTAATTTACTCTTTCAATTTTGTAATATTTGGTTACATTTCTCGTTTTCAT
HM1-1     AATCTTTGACTAATTTACTCTTTCAATTTTGTAATATTTGGTTACATTTCTCGTTTTCAT
```

```
DHL92          TTTCTTTGACTAATTTACTCTTTCAATTTTGTAATATTTGGTTACATTTCTCGTTTTCAT
                ***********************************************************

ms-5           TATGATATCTTTGTCTCTTTTATTTTGGTAAAATCTTTTTGATAGAACTATGTTGAAAGT
HM1-1          TATGATATCTTTGTCTCTTTTATTTTGGTAAAATCTTTTTGATAGAACTATGTTGAAAGT
DHL92          TATGATATCTTTGTCTCTTTTATTTTGGTAAAATCTTTTTGATAGAACTATGTTGAAAGT
               ************************************************************

ms-5           ATCTCTTCTTTTTTTTTTTTTTTCTCTCTATTTTTTTAAATAGTAGACCTTTTTTTTTTT
HM1-1          ATCTTTTTTTTTTTTTTTTTTTCTCTCTCTATTTTTTTAAATAGTAGANNTTTTTTTTTTT
DHL92          ATCTCTTCTTTTTTTTTTTTTTTCTCTCTATTTTTTTAAATAGTAGACCTTTTTTTTTTT
               **** ** ** ******** * *************************  ***********

ms-5           CTTTTTCTATAATGGTAGACATTTTCTCCAATTTTTCTTACATTTGTGGTACATCTTTTT
HM1-1          NTTTTTCTATAATGGTAGACATTTTCTCCAATTTTTCTTACATTTGTGGTACATCTTTTT
DHL92          CTTTTTCTATAATGGTAGACATTTTCTCCAATTTTTCTTACATTTGTGGTACATCTTTTT
               ************************************************************

ms-5           TTTTCACGTCTTTAAACTATTTTTCTTGTTTAAAATTCTAACCTTATATTTTTTACCATT
HM1-1          TTTTCACGTCTTTAAACTATTTTTCTTGTTTAAAATTCTAACCTTATATTTTTTACCATT
DHL92          TTTTCACGTCTTTAAACTATTTTTCTTGTTTAAAATTCTAACCTTATATTTTTTACCATT
               ************************************************************

ms-5           TTTCATTCTCAGATTGAGAGTTAAATTAAAA
HM1-1          TTTCATTCTCAGATTGAGATTTAAATTAAAA
DHL92          TTTCATTCTCAGATTGAGAGTTAAATTAAAA
               ******************* ***********
```
